# Crescentic poststreptococcal acute glomerulonephritis accompanied by small vessel vasculitis: case report of an elderly male

**DOI:** 10.1186/s12882-019-1663-9

**Published:** 2019-12-18

**Authors:** Keiko Yano, Hiroyuki Suzuki, Takashi Oda, Yoshihiko Ueda, Tatsuo Tsukamoto, Eri Muso

**Affiliations:** 1 Department of Nephrology and Dialysis, Kitano Hospital, Tazuke Kofukai Medical Research Institute, 2-4-20 Ohgimachi, Kita-ku, Osaka, 530-8480 Japan; 2 Department of Nephrology, Graduate School of Medicine, Kyoto University, 54 Shogoin Kawahara-cho, Sakyo-ku, Kyoto, 606-8507 Japan; 3 Department of Nephrology and Blood Purification, Kidney Disease Center, Tokyo Medical University Hachioji Medical Center, 1163 Tatemachi, Hachioji, Tokyo, 193-0998 Japan; 4 Department of Pathology, Dokkyo Medical University Saitama Medical Center, 2-1-50 Minami Koshigaya, Koshigaya, Saitama, 343-8555 Japan; 5 Department of Food and Nutrition, Faculty of Contemporary Home Economics, Kyoto Kacho University, 3-456 Rinka-cho, Higashiyama-ku, Kyoto, 605-0062 Japan

**Keywords:** Poststreptococcal acute glomerulonephritis, Anti-neutrophil cytoplasmic antibody-associated vasculitis, Peritubular capillaritis, Hilar arteriolitis, Nephritis-associated plasmin receptor

## Abstract

**Background:**

Poststreptococcal acute glomerulonephritis (PSAGN) in the elderly tends to have a severe clinical course and often presents with crescentic necrotizing glomerulonephritis in the renal biopsy. However, vasculitis lesions are unusual.

**Case presentation:**

We present a 71-year-old man who was admitted to our hospital for a recurrent gout attack with a rapid decline of renal function. Low C3 levels and a high anti-streptolysin O titer were observed, while myeloperoxidase- and proteinase 3- antineutrophil cytoplasmic antibody (ANCA) were negative. In addition to cellular crescent and necrosis lesions, diffuse peritubular capillaritis and venulitis as well as small arteriole vasculitis in the glomerular hilus were also apparent. Although granular C3c deposits in the capillary wall and hump lesions were not found, immunofluorescent staining for nephritis-associated plasmin receptor (NAPlr) and in situ zymography for plasmin activity were both positive. We thus diagnosed PSAGN accompanied by small vessel vasculitis. Steroid therapy gradually improved the patient’s renal function, and hemodialysis was discontinued after 1 month.

**Conclusions:**

In our case, streptococcus infection might have concurrently provoked vasculitis, and NAPlr staining was useful for confirming diagnosis.

## Background

Poststreptococcal acute glomerulonephritis (PSAGN) in the elderly tends to have a poor prognosis. In severe cases, cellular crescents and necrosis are observed in addition to endocapillary hypercellularity in the affected glomeruli; however, vasculitis lesions are rare. Here we report a severe case of PSAGN that developed rapidly progressive glomerulonephritis and required renal replacement therapy. In addition to the pathological findings described above, diffuse peritubular capillaritis (PTCitis) and venulitis as well as arteriolitis at the glomerular hilus were also observed. Although these findings were consistent with concurrent small vessel vasculitis, antineutrophil cytoplasmic antibody (ANCA) was not detected in our case. Intensive treatment, including glucocorticoid, resulted in the successful recovery of the patient’s renal function.

## Case presentation

A 71-year-old Japanese man was admitted to our hospital because of severe pain and swelling of the right ankle. He had a past history of recurrent gout arthritis and diabetes mellitus. He used non-steroidal anti-inflammatory drug (NSAIDs), such as diclofenac suppositories and loxoprofen, together with daily glimepiride, metformin, benzbromarone and potassium citrate-sodium citrate hydrate. On admission, his body temperature was 37.1 °C and blood pressure was 140/75 mmHg. Bilateral lower leg edema and swollen right ankle and knee were observed. Skin lesions, such as purpura, were not observed. He did not present with symptoms of skin or throat infection; however, it is unknown whether he had a past infection before admission. Laboratory tests revealed high levels of white blood cells (14,300/μL) and C-reactive protein (15.92 mg/dL). Unexpectedly, an elevated creatinine level (3.28 mg/dL, increased from 1.16 mg/dL within a week) was detected. On the next day, he presented with oliguria. Since there was no response to diuretic drugs and his creatinine had increased to 5.81 mg/dL, hemodialysis was started on day 3.

**Fig. 1:**
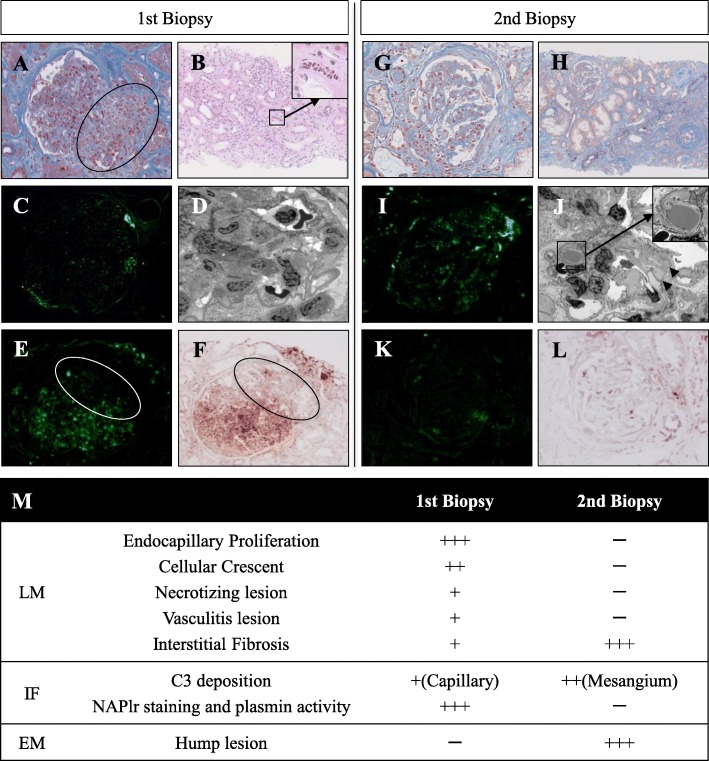
Findings in the first (**a**-**f**) and second biopsy (**g**-**l**), which is summarized in **m**. **a** Prominent endocapillary hypercellularity with infiltrating leukocytes. Arteriolitis in the glomerular hilus *(indicated by circle)* (El-M staining, magnification × 400). **b** Neutrophil infiltration and fibrosis in the interstitium*.* PTCitis included abundant neutrophils *(indicated and zoomed by box)* (HE staining, magnification × 100). **c** Weak fine granular C3c deposition was observed in the capillary wall. **d** Diffuse endocapillary proliferation and mesangiolysis was observed. Neither hump lesions nor apparent electron dense deposit was observed. Foot process effacement of the epithelial cells was globally observed (magnification × 2000). **e** NAPlr deposition was observed in the mesangium, subendothelial area and neutrophils. **f** Plasmin activity was distributed the same as in **e**. Both findings in **e** and **f** were limited in the tuft area and negative at the arteriole in hilus of glomerulus (*indicated by circle*). **g** Disappearance of endocapillary proliferation. Shrinking and double contour of capillary wall was globally apparent (El-M staining, magnification × 200). **h** Massive diffuse interstitial fibrosis and tubular atrophy. There were no findings of active arteritis. (El-M staining, magnification × 40). **i** C3c deposition was observed predominantly in the mesangial area. **j** Large subepithelial electron-dense deposits regarded as hump lesions were observed with various densities (*indicated by arrowhead*). None of them contained an organized structure (*indicated and zoomed by box*) (magnification × 1500). **k** NAPlr deposition was faded out and localized segmentally. **l** Plasmin activity was faded out the same as in **k**. **m** Summary of pathologic findings. HE: Hematoxylin eosin, PAM: Periodic acid-methenamine silver, PAS: Periodic acid-Schiff, El-M: Elastic-Masson

Details of the laboratory tests are shown in the Additional file 1: Table S1. Low levels of C3 were detected. Myeloperoxidase- and proteinase 3-ANCA were negative. Culture tests of blood, urine and joint fluid were negative for infection. Instead, paracentesis revealed the abnormal formation of calcium pyrophosphate crystals, which suggested the diagnosis of pseudogout. His joint pain promptly improved by steroid injection. We did not use antibiotics since there was no evidence of concurrent infection. NSAIDs-associated nephropathy was initially suspected, yet his renal function did not show improvement. While providing symptomatic treatment, we performed a renal biopsy on day 7. Twenty-six glomeruli, including ten global scleroses, were obtained. The remaining glomeruli showed endocapillary hypercellularity, infiltrating polymorphonuclear neutrophils, with mild mesangial proliferation. Cellular crescents were found in six glomeruli, and some accompanying necrotizing lesions. In addition, diffuse PTCitis and venulitis as well as arteriolitis at the glomerular hilus were prominent (Fig. 1a, b). Focal neutrophil infiltration, including a few eosinophils were also observed in tubulointerstitium. Immunofluorescent (IF) study showed weak fine granular C3c deposition in the capillary wall (Fig. 1c) without any immunoglobulin including IgA. Positive anti-streptolysin O (ASO) (512 U/mL, reference value: ≦160 U/mL) on day 12 suggested a diagnosis of PSAGN. However, neither hump lesions nor glomerular electron-dense deposit (EDD) were evident, although global foot process effacement was observed in electron microscopy (Fig. 1d). Therefore, we further examined the pathogenesis by IF staining for nephritis-associated plasmin receptor (NAPlr) and in situ zymography for plasmin activity using our methods as previously reported [1], both of which were strongly positive. It should be noted that the positivity of these factors was limited to the glomerular tuft and not detected in arteriole at the glomerular hilus, which suggest a distinct pathogenesis of vasculitis from glomerulitis. (Fig. 1e, f). Thus, we diagnosed PSAGN combined with small vessel vasculitis. Since a few eosinophils were observed in tubulointerstitium, drug-induced kidney injury especially NSAID-induced allergic interstitial nephritis (AIN) could not be ruled out. However, neutrophils were much abundant, it was conceivable that inflammatory cell infiltration was associated with PTCitis.

**Fig. 2:**
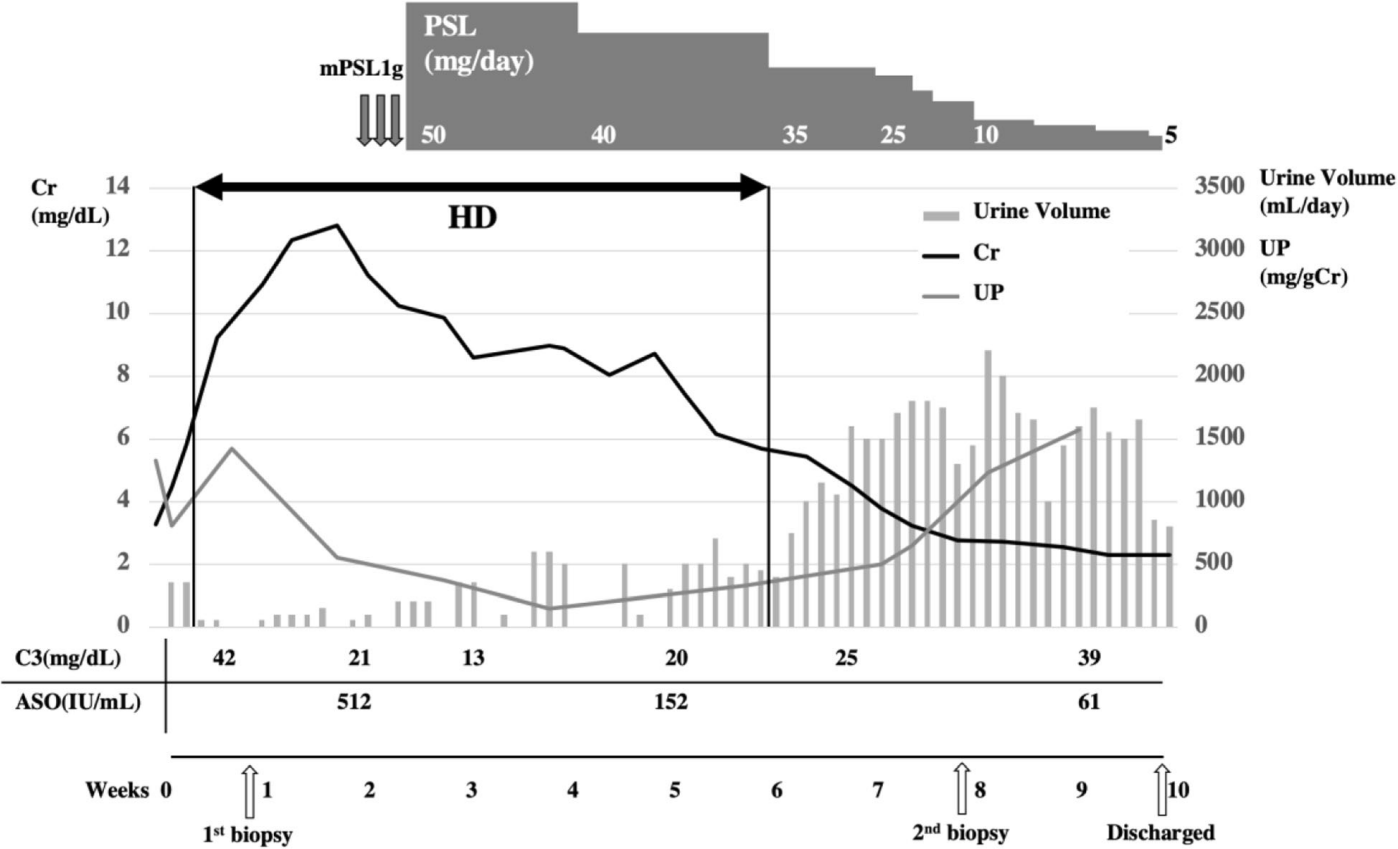
Clinical course of the patient. Changes in the serum creatinine (Cr, black lines), urine protein (UP, gray lines), urine volume (gray bars), C3 and ASO. HD: Hemodialysis, mPSL: Methylprednisolone, PSL: Prednisolone, UP: Urine protein, Cr: Serum creatinine, ASO: Anti-streptolysin O antibody

The patient’s clinical course is depicted in Fig. 2. Intravenous methylprednisolone pulse therapy (1 g/day) followed by oral prednisolone (50 mg/day) was started after the pathological diagnosis ruled out NSAIDs-induced AIN. Since the diagnosis of PSAGN was 2 weeks later from the onset and there was no evidence of persistent streptococcus infection, we did not prescribe antibiotics. His kidney function gradually improved, and hemodialysis was discontinued on day 48. We performed a follow-up renal biopsy. Eleven glomeruli with eight global scleroses were obtained. The remaining glomeruli were also collapsing, yet crescent formation and endocapillary proliferation were no longer present (Fig. 1g, h). PTCitis had disappeared, but diffuse interstitial fibrosis and tubular atrophy were observed. IF study showed C3c deposition in the mesangium and the capillary wall (Fig. 1i). Both NAPlr staining and plasmin activity were faded out whereas various hump lesions were evident (Fig. 1j-l), indicating the recovery phase of PSAGN. We reduced prednisolone immediately to 5 mg/day, and the patient was discharged on day 67. His final creatinine level was 2.2 mg/dL, and his C3 level normalized 4 months after disease onset.

## Conclusion

We reported an elderly case of severe PSAGN accompanied with small vessel vasculitis. Streptococcus is a common bacterial agent in infection-related glomerulonephritis (IRGN) for both children and adults, with an increased morbidity in the elderly. Nasr examined 109 cases of IRGN patients over 65 years of age. The mean serum creatinine level at the first visit increased to 5.1 mg/dL, and 32 patients (46%) required renal replacement therapy. Of these, 60% of patients were immunocompromised as a result of diabetes, malignant tumor or alcohol abuse [2]. These backgrounds might not only increase the susceptibility of IRGN but also exacerbate the disease [3].

In our case it was difficult to diagnose PSAGN for two reasons. First, there was a lack of clear findings of preceding infection. Most cases of PSAGN develop about 2 weeks after upper respiratory tract or skin infections. Our patient, however, had neither a past history nor evidence of infection on admission [2, 4, 5]. Second, EDDs including hump lesions, which are compatible with faint C3c staining, were absent in the first biopsy. Moreover, although an elevated ASO titer may be supportive, it does not confirm the diagnosis of PSAGN [6]. Therefore, we employed NAPlr staining to confirm our diagnosis. NAPlr is a nephritogenic agent isolated from group A streptococcus that is homologous to streptococcal glyceraldehyde-3-phosphate dehydrogenase. NAPlr can bind with and maintain the proteolytic activity of plasmin, which could accumulate inflammatory cells and enhance the permeability of the glomerular basement membrane. NAPlr may participate in immune complex formation in the circulating blood stream or in situ to form hump lesions [7, 8]. Yamakami reported the glomerular NAPlr deposition in 100% of renal tissues from PSAGN patients biopsied within 2 weeks of disease onset. On the other hand, NAPlr deposition was found in only 4% of other glomerulonephritis and was not observed in normal kidneys [9]. Furthermore, hump lesions become evident in the later phase since they are formed as an accumulation of immune complexes, complements and plasma protein [10]. Indeed, massive hump lesions were observed while NAPlr and plasmin activity faded out in the second biopsy. Thus, NAPlr staining appears to be useful for supporting the diagnosis of PSAGN in hyperacute-phase and hump-negative cases.

Vasculitis features are usually unremarkable in PSAGN. Previously, Bodaghi reported four children with vasculitis in PSAGN as a rare case [11]. Even in IRGN, vasculitis and fibrinoid necrosis are only found in 1 to 6% of cases [3, 4]. ANCA-associated vasculitis (AAV) must be considered, especially in elderly patients, because the prevalence dramatically increases over the age of 50. Indeed, Ardiles reported that 9% of PSAGN cases were ANCA-positive [12]. However, our case was ANCA negative in spite of diffuse PTCitis, venulitis and arteriolitis. We summarized 3 case reports of PSAGN accompanied with vasculitis lesion which was ANCA-negative in Table 1 [13–15]. There was a very similar case report of a 12-year-old boy diagnosed with ANCA-negative vasculitis accompanied by streptococcus infection that also revealed positive NAPlr staining in glomeruli [13]. The pathogenesis is unclear; however, streptococcus infection might have induced vasculitis via neutrophil activation. During the process of neutrophil activation and cell death, neutrophil extracellular traps (NETs) are released which consists of DNA backbones and various proinflammatory proteins. NETs can not only trap and kill the microorganisms, but also activate other immune cells and increase the inflammation. Recent studies have proven that NETs provoke inflammation and result in a vicious cycle of AAV [16]; therefore, it is easily speculated that a similar background was present in our case.

**Table 1:** Clinical profiles and pathological findings of PSAGN with “ANCA-negative” vasculitis

Age/Sex	Cre (mg/dL)	Alb (g/dL)	WBC (/μL)	CRP (mg/dL)	C3 (mg/dL)	C4 (mg/dL)	ASO (IU/mL)	Culture test (specimen)	Onset to biopsy (day)	Crescent formation	Interstitial Inflammation	Vasculitis lesion	IF	hump	NAPlr staining	Treatment	Reference
71/M	3.28	3.6	14,300	15.92	42	27	520	negative (blood, urine, joint fluid)	7	+	+	PTCitis, venulitis, arteriolitis in the glomerular hilus	no deposition	–	+ (glomeruli)	corticosteroids hemodialysis	Our case
12/M	3.15	1.92	24,600	15.74	103	14	101	negative (blood)	13	–	+	Fibrinoid necrosis in arteriole	no deposition	–	+ (glomeruli)	corticosteroids hemodialysis antibiotics	[13]
72/M	7.92	NA^a^	NA	NA	59	26	340	NA	NA	–	+	PTCitis	C3 (capillary, mesangium)	NA	NA	corticosteroids hemodialysis	[14]
53/M	11.8	NA	NA	> 15.00	41	7	1150	negative (synovial fluid, throat)	NA	–	+	Fibrinoid necrosis, hyaline thrombi in arteriole	IgG, C3 (capillary)	+	NA	corticosteroids antibiotics	[15]

^a^*NA* Not available

Ultimately, we used glucocorticoids, and successfully induced a decrease in disease activity and enabled the discontinuation of hemodialysis. Immunosuppressive therapy, such as steroids and calcineurin inhibitors, are not generally recommended for PSAGN even for pediatric patients [5]. However, several reports have suggested that aggressive therapy would be effective for severe PSAGN cases that present with cellular crescent formation and diffuse interstitial infiltration of inflammatory cells [17]. Glassock emphasized that clinicians should focus on the important differences between true “post”-infectious glomerulonephritis, such as PSAGN, and other IRGN, such as IgA-dominant lesions associated with “ongoing” staphylococcal infection [18]. In other words, PSAGN is characterized as an immune-mediated disease, and streptococcus infection is just the cause. Therefore, immunosuppressive therapy should be considered in severe cases to suppress the autoimmune reaction. We did not use other immunosuppressive drugs such as cyclophosphamide or rituximab, which are commonly recommended in AAV, mainly because the response to the steroid was obtained in a relatively early phase and also because the main cause of the deterioration of renal function was suggested to be due to massive PSGN for which the benefit of such immunosuppressants has not been widely proven.

In conclusion, NAPlr staining is useful for confirming the diagnosis of PSAGN. We also emphasize that the association of vasculitis must be considered in severe cases, especially for elderly patients. It is important to make a precise pathological diagnosis and to provide aggressive immunosuppressive therapy to prevent progression to end-stage renal disease.

## Supplementary information

**Additional file 1:** **Table S1.** Laboratory data (Admission day)

## Data Availability

The datasets during this case report are available from the corresponding author on reasonable request.

## Abbreviations

AAV: ANCA-associated vasculitis
AIN: Allergic interstitial nephritis
ANCA: Antineutrophil cytoplasmic antibody
ASO: Anti-streptolysin O
IRGN: Infection-related glomerulonephritis
NAPlr: Nephritis-associated plasmin receptor
NETs: Neutrophil extracellular traps
PSAGN: Poststreptococcal acute glomerulonephritis
PTCitis: Peritubular capillaritis
