# Supplementary material for: Crescentic poststreptococcal acute glomerulonephritis accompanied by small vessel vasculitis: case report of an elderly male
Source: BMC Nephrol. 2019 Dec 18;20:471. doi: 10.1186/s12882-019-1663-9 (PMC6921464; doi:10.1186/s12882-019-1663-9)
Supplement: Supplementary file 1 — Additional file 1: Table S1. Laboratory data (Admission day) [file 12882_2019_1663_MOESM1_ESM.docx]

**Additional file 1: Table S1**

Laboratory data (Admission day)

| Urinalysis | | | Blood chemistry | | | Serological test | | |
| --- | --- | --- | --- | --- | --- | --- | --- | --- |
| Specific gravity | 1.016 |  | AST | 18 | IU/L | IgG | 1480 | mg/dL |
| pH | 5.5 |  | ALT | 17 | IU/L | IgA | 388 | mg/dL |
| Protein | (3+) |  | LDH | 227 | IU/L | IgM | 46 | mg/dL |
|  | 1.3 | g/gCre | ALP | 330 | IU/L | C3 | 42 | mg/dL |
| Glucose | (-) |  | γ-GTP | 144 | IU/L | C4 | 27 | mg/dL |
| Occult blood | (3+) |  | TP | 7.1 | g/dL | ANA | (-) |  |
| RBC | 50-99 | /HPF | Alb | 3.6 | g/dL | dsDNA Ab | (-) |  |
| WBC | >100 | /HPF | Glu | 199 | mg/dL | PR3-ANCA | (-) |  |
| Granular casts | 1-4 | /LPF | T-Chol | 204 | mg/dL | MPO-ANCA | (-) |  |
| WBC casts | 1-4 | /LPF | LDL-C | 127 | mg/dL | Anti-GBM Ab | (-) |  |
| RBC casts | 1-4 | /LPF | BUN | 68 | mg/dL | Cryoglobulin | (-) |  |
| NAG | 41.2 | U/L | Cre | 3.28 | mg/dL | HBs Ag | (-) |  |
| β2MG | 76 | μg/L | UA | 10.7 | mg/dL | HCV Ab | (-) |  |
|  |  |  | Cr | 3.28 | mg/dL | HIV Ab | (-) |  |
| CBC | | | Na | 139 | mEq/L |  |  |  |
| WBC | 14300 | /μL | K | 5.2 | mEq/L |  |  |  |
| Neut | 80.5 | % | Cl | 108 | mEq/L |  |  |  |
| Ly | 7.8 | % | Ca | 9.2 | mg/dL |  |  |  |
| Mono | 11.5 | % | P | 4.5 | mg/dL |  |  |  |
| Eo | 0.1 | % | CRP | 15.92 | mg/dL |  |  |  |
| RBC | 347x10^4^ | /μL | HbA1c | 8.0 | % |  |  |  |
| Hb | 12 | g/dL |  |  |  |  |  |  |
| Ht | 35.7 | % |  |  |  |  |  |  |
| Plt | 32.0x10^4^ | /μL |  |  |  |  |  |  |

Conversion factors for units: T-Chol and LDL-C in mg/dL to mmol/L, ×0.02586; UA in mg/dL to μmol/L, ×59.48; Ca in mg/dL to mmol/L, ×0.2495; BUN in mg/dL to mmol/L, ×0.357; Cr in mg/dL to μmol/L, ×88.4.
